# The Medical Research Council Myeloma IX trial: the impact on treatment paradigms*

**DOI:** 10.1111/j.1600-0609.2011.01721.x

**Published:** 2012-01

**Authors:** Paul G Richardson, Jacob P Laubach, Robert L Schlossman, Irene M Ghobrial, Constantine S Mitsiades, Jacalyn Rosenblatt, Anuj Mahindra, Noopur Raje, Nikhil Munshi, Kenneth C Anderson

**Affiliations:** 1 Department of Medical Oncology, Dana-Farber Cancer InstituteBoston, MA; 2 Beth Israel Deaconess Medical CenterBoston, MA; 3 Division of Hematology-Oncology, Harvard Medical SchoolBoston, MA, USA

**Keywords:** bisphosphonate, clodronate, multiple myeloma, pamidronate, zoledronic acid

## Abstract

Osteolytic bone disease is a hallmark of symptomatic multiple myeloma. Bisphosphonates have been the mainstay of treatment to preserve skeletal integrity and prevent skeletal-related events in patients with myeloma-related bone disease. Recently, the MRC Myeloma IX trial demonstrated for the first time improved survival and delayed disease progression with the use of an intravenous amino-bisphosphonate, zoledronic acid, vs. an oral agent, clodronate, with intensive and non-intensive anti-myeloma treatment regimens in patients with newly diagnosed multiple myeloma. These results validate a large body of preclinical, translational and other clinical data suggesting anti-myeloma effects of amino-bisphosphonates. In addition, this trial also provided the first head-to-head evidence for superiority of one bisphosphonate over another (zoledronic acid vs. clodronate) for reducing skeletal morbidity in patients with multiple myeloma, as well as a prospective comparison of toxicities. Despite the use of non-bortezomib containing anti-myeloma treatment regimens in the MRC Myeloma IX trial, these results are encouraging and provide an impetus to continue to evaluate current treatment guidelines for myeloma-associated bone disease.

## Introduction

Approximately 100 000 new cases of multiple myeloma (MM) are diagnosed each year worldwide (1), and MM accounts for 1% of all cancer-related deaths (∼72 000 deaths annually) (1). Survival for patients with MM can range from <6 months to more than 10 yr based on disease stage and prognostic factors (2). Clonal expansion of malignant, terminally differentiated, B-lymphocyte–derived plasma cells is characteristic of MM and typically results in excessive production of monoclonal immunoglobulins, thereby contributing to disruption of immunologic activity and contributing to renal failure as well as other complications, such as hyper viscosity (3, 4). Moreover, this neoplastic plasma cell expansion with its attendant effects on the cytokine milieu disrupts normal haematopoiesis (leading to anaemia) and skeletal homoeostasis (resulting in extensive osteolytic lesions). As a consequence, serum calcium levels may be elevated and patients can develop debilitating skeletal-related events (SREs; including pathologic fracture, spinal cord compression and bone pain requiring surgery or palliative radiotherapy).

The severity of bone lesions and levels of haemoglobin, serum calcium, serum creatinine, C-reactive protein (CRP), serum albumin and β_2_-microglobulin (β_2_M) have been identified as independent prognostic factors for survival in patients with MM, and have been incorporated into staging systems such as the Durie-Salmon (5) and International Staging Systems (ISS) (2). The widely used ISS is a 3-stage classification of MM that uses serum β_2_M and albumin levels for prognostication. New treatment options for MM have greatly improved survival rates, with median survival exceeding 5 yrs for patients with ISS stage I disease (2), and reports of survival exceeding 10 yrs in some patients with advanced disease undergoing stem-cell transplant and/or receiving novel anti-myeloma regimens (2). However, outcomes have typically been poor for patients with high-risk disease (e.g. median survival ∼6 months in patients with high levels of CRP and β_2_M, vs. ∼54 months in patients with low levels of these factors) (6) and, despite recent therapeutic advances, the outlook for such patients remains guarded.

## Pathophysiology of myeloma bone disease

Myeloma-bone interactions typically result in increased rates of osteoclast-mediated osteolysis, and myeloma cells can secrete factors that inhibit osteoblast function (osteogenesis) (7). Myeloma cells in the bone microenvironment typically secrete factors that interact with and influence release of bone marrow-derived growth factors and signalling intermediates, thereby rendering the bone marrow even more conducive to myeloma growth, and potentially setting up a cycle of osteolysis and myeloma cell proliferation (7). In addition, myeloma cells also stimulate secretion of receptor activator of nuclear factor-kappa B (NF-κB) ligand (RANKL) and inhibit expression of osteoprotegerin (OPG; the decoy receptor for RANKL) by osteoblasts, resulting in localised promotion of bone resorption by osteoclasts to levels that greatly exceed compensatory bone formation by osteoblasts which in turn are suppressed by humoral factors such as dickkopf 1 (DKK1) (7). Consequently, bone lesions from MM are highly destructive, and appear on radiographs as purely lytic areas of ‘punched-out bone’, which is quite different from the radiographic appearance of osteolytic and sclerotic metastases from most solid tumours. Low bone-mineral density and osteoporotic fractures are also common among patients with MM (8, 9) and may often be underdiagnosed, thereby increasing the risk of extensive bone damage before appropriate therapeutic intervention follows (8).

## Supportive treatment for symptomatic disease

Symptomatic MM is typically characterised by elevated serum calcium levels, renal deterioration, anaemia and bone disease: a cluster of clinical manifestations often referred to as CRAB criteria. Of these features, elevated serum calcium and bone disease (specifically, SREs) are readily addressed by therapeutic intervention with bisphosphonates (BPs). Over the last 15 yrs, the efficacy of BP therapy in preserving skeletal health and mitigating SRE risk in patients with MM has been well established. Accordingly, BPs have been incorporated as a supportive therapy in patients with MM. Current American Society of Clinical Oncology (ASCO) guidelines recommend intervention with BP therapy for 2 yrs in patients with MM with radiographic evidence of bone lysis or compression fracture (10). The National Comprehensive Cancer Network (NCCN) recommendations are similar, although they do not clearly specify the duration of BP therapy (11). It should be noted that caution is advised with use of BPs, given the incidence of certain complications, including osteonecrosis of the jaw (ONJ) (10).

**Figure 1:**
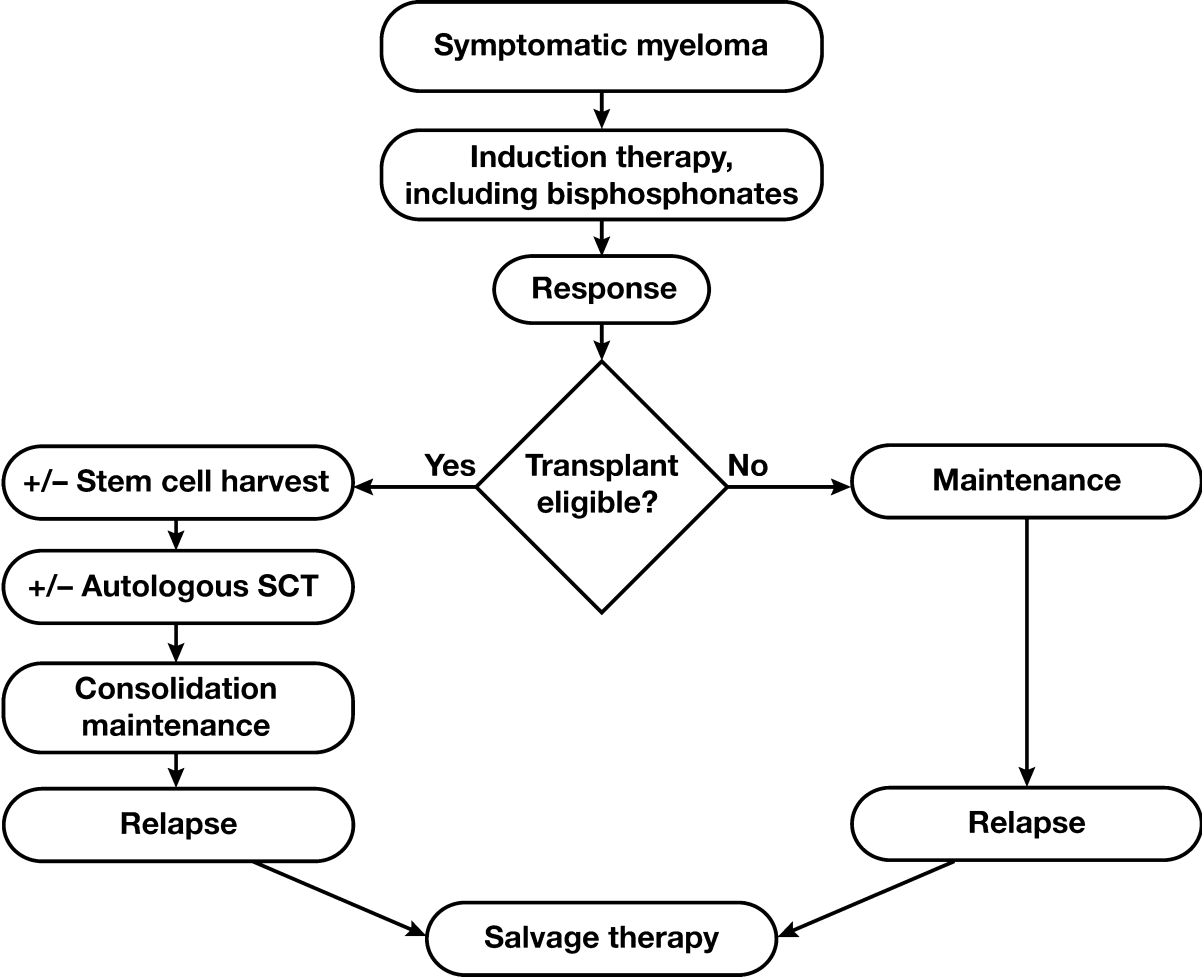
Treatment pathways in patients with newly diagnosed symptomatic multiple myeloma. SCT, stem cell transplant.

Recently, newer agents have vastly improved clinical outcomes for patients with symptomatic MM, and although the treatment of MM has evolved in many respects, the overall schema remains relatively unchanged, although this may vary between the United States and Europe (12) (Fig. 1). Patients with MM typically undergo one of two main treatment pathways based on the feasibility and estimated benefit of haematopoietic stem cell transplant, although the necessity of this approach in all younger patients is now an area of active research. Although younger and fitter patients with good performance status are candidates for stem cell transplant, older patients and those with poor performance status may not derive benefit. In both cases, patients typically receive systemic therapy consisting of an induction/consolidation and a maintenance phase. As mentioned, supplementary BP therapy to preserve skeletal health is indicated for patients with symptomatic MM and should be considered in all newly diagnosed patients in whom there is no known contraindication.

## Can bisphosphonates do more than strengthen bone?

The development and progression of MM within the bone are mediated by signalling of adhesion molecules and the subsequent activation/secretion of cytokines and growth factors that promote a destructive cycle of myeloma growth and bone loss (13). In BP-treated patients, the drug is rapidly bound to the bone, where it is taken up by both osteoclast and non-osteoclast cells, modifying their function, intracellular interactions and cellular fate (14). The coincident overlap of location and cellular/molecular components involved in MM and targeted by BPs may disrupt or modify the interactions of myeloma cells with the bone microenvironment to inhibit MM progression. Moreover, as BPs are rapidly cleared from systemic circulation, they have minimal pharmacokinetic interaction with other therapies and may be combined with various anti-neoplastic agents, as has been confirmed in several studies (15). Certainly, both preclinical and translational evidence supports potential anti-myeloma effects of BPs; that is, increased host immunity, overcoming resistance, and potential synergy with other agents.

Preclinical data suggest single-agent anti-myeloma activity for BPs (including zoledronic acid [ZOL]), as well as at least additive activity with anti-myeloma agents including thalidomide, dexamethasone and interleukin-6 antagonists in MM models (16–22). In addition, additive and/or synergistic activity has been reported with combinations of ZOL and imatinib (23), hydroxyurea, cytarabine and daunorubicin (24) in leukaemia cell lines. Preclinical models of MM also suggest that nitrogen-containing BPs (e.g. ZOL and pamidronate [PAM]) may induce myeloma cell apoptosis, inhibit disease progression and prolong survival (19, 22) specifically via their inhibitory effects on the mevalonate pathway (resulting in impaired protein prenylation, signalling and consequently cell viability and function) (22). Some of these preclinical mechanisms support observations from translational and pilot studies. For example, in one study, increased levels of apoptotic plasma cells were detected in bone marrow aspirates from 14 of 16 patients with newly diagnosed MM after a single infusion of PAM (90 mg) (25). An added consequence of inhibiting the mevalonate pathway is the accumulation of isopentenyl pyrophosphate (IPP), an intermediate implicated in the activation and expansion of a subset of T cells (Vγ9Vδ2; which exert anti-cancer immune activity) in blood samples from MM patients (26). Potential anti-myeloma effects of ZOL have also been reported in pilot studies in patients with symptomatic (27), but not smouldering MM (28). However, until recently, the clinical benefit of BPs had not been prospectively and systematically explored in patients with newly diagnosed MM in both the stem cell transplant and non-stem cell transplant settings.

The large, independent, randomised and prospectively controlled Phase III MRC Myeloma IX trial compared the relative efficacy of zoledronic acid (ZOL) vs. clodronate (CLO) (*N* = 1960) for reducing SREs and improving disease-related outcomes across the prevailing standard treatments in patients with newly diagnosed, symptomatic MM (29). The primary efficacy endpoints assessed in this trial included progression-free survival, overall response rate and overall survival (OS). Secondary endpoints included SRE incidence and toxicity.

Most patients had documented myeloma bone disease (∼70%) at study entry. Zoledronic acid significantly prolonged both progression-free survival and OS (*P* = 0.0179 and *P* = 0.0118, respectively) vs. CLO. Moreover, the OS curves showed an early (within 4 months) and sustained separation between the ZOL and CLO arms, suggestive of benefit to patients treated with ZOL. Zoledronic acid also reduced the proportion of patients with an SRE vs. CLO (27.0% vs. 35.3%, respectively; *P* = 0.0004). It should be noted that the improvement in OS was maintained after adjustment for time to first SRE in a Cox model (*P* = 0.0178), further suggesting that ZOL-mediated anti-myeloma effects likely underlie the OS benefit. Among patients allocated to the non-intensive pathway, ZOL treatment significantly improved the complete or very-good-partial response rate (*P* = 0.03) (29). In contrast, ZOL did not significantly improve the response rate in patients allocated to the intensive pathway, perhaps because of the higher overall response rate among patients undergoing myeloablative therapy. Overall, the MRC Myeloma IX study provides evidence for an anti-myeloma effect of ZOL over and above that provided by CLO, which had also previously demonstrated OS benefit vs. placebo, albeit restricted to the subset of patients without skeletal fractures at presentation (30).

These data are concordant with previous clinical data that suggest BPs may provide an anti-myeloma benefit, at least within certain subsets of patients. For example, long-term treatment with intravenous PAM significantly increased survival in the subset of patients with MM receiving second-line anti-myeloma therapy (*n* = 130; 14 vs. 21 months; *P* = 0.041) compared with placebo (31). Similarly, in a retrospective analysis of 353 patients with bone lesions with MM, ZOL treatment prolonged OS in patients with high bone turnover (subset of patients with high bone alkaline phosphatase levels, *n* = 89), compared with PAM (32). More recently, Aviles *et al.* (27) showed that combining ZOL with conventional chemotherapy in treatment-naive patients (*N* = 94) significantly improved 5-yr event-free survival (80% vs. 52%, respectively) and 5-yr overall survival (80% vs. 46%, respectively; *P* < 0.01 for both) compared with conventional therapy alone. In contrast, the addition of PAM to thalidomide for maintenance treatment of patients with MM did not confer a survival advantage (33). However, in this study, PAM treatment may have been suboptimal, as suggested by the lack of significant effect observed on SRE incidence (*P* = 0.4).

The most recent Cochrane systematic review of BPs in MM concluded that BP treatment was not associated with improved survival among MM patients (34). Typically, in these types of analyses, effects on particular patient subsets and activity of particular BPs may be masked, and as noted by the authors, there was significant heterogeneity among these trials. It is also important to note that this Cochrane analysis (34) pre-dates the release of the MRC Myeloma IX data. Reflective of this, an updated analysis by the same group, presented at the American Society of Hematology annual meeting, demonstrated superiority of ZOL over other BPs for improving OS and potentially also preventing SREs in patients with MM (35).

It should be noted that ZOL differs from early generation agents such as CLO in terms of both mechanism of action and effectiveness in inhibiting bone resorption. Newer-generation BPs such as ZOL are more effective inhibitors of bone resorption and potentially are able to demonstrate greater anti-myeloma activity (14, 15). In addition, clinical data show that nitrogen-containing BPs such as ZOL may inhibit tumour progression by enhancing host anti-cancer immune response and inhibiting tumour-mediated angiogenesis (15). Thus, it is perhaps not surprising that ZOL improved myeloma-related outcomes compared with CLO in the MRC Myeloma IX study.

Overall, in the MRC Myeloma IX trial, ZOL was generally well tolerated with a small proportion of patients (11–14%) still receiving BP after 4 yrs on study. Early deaths (within the first 4 months) attributed to infection and renal failure occurred more frequently among patients treated with CLO compared with ZOL. The overall incidence of confirmed ONJ among ZOL-treated patients was significantly higher than in patients treated with CLO (3.6% vs. <1%, respectively; *P* < 0.001) (29). Implementation of preventive measures, as done in this trial, may reduce the incidence of ONJ (10). Interestingly, there was no significant difference in the incidence of drug-related renal toxicity between study arms. Taken together, the clinical benefit provided by ZOL appears to outweigh the risk of ONJ, especially if appropriate precautions are taken.

The MRC Myeloma IX trial has certain limitations. The study was not prospectively designed to explore translational endpoints (e.g. serum cytokine/growth factor levels) that might provide insights into the potential anti-MM activity of ZOL. Such studies not only provide proof of principle, but may also provide additional information on future combinations of agents as treatment regimens, and agents continue to evolve. Moreover, although the survival benefits from adding ZOL to the standard therapies used in MRC Myeloma IX appeared to be broadly independent of treatment pathway, current bortezomib-based standards of care were not included in this study, as the study preceded the emergence of this key agent as a standard of care in the United Kingdom. Existing data suggest a synergy between ZOL and bortezomib in preventing bone resorption, including new bone formation through osteoblast activation and inhibiting myeloma progression; their combination may therefore enhance clinical benefit (36, 37).

## Will the MRC Myeloma IX results alter treatment guidelines for MM?

Overall, the results from MRC Myeloma IX support the use of ZOL therapy in newly diagnosed MM patients with early bone disease. In addition, these data support previous observations from prior clinical studies suggestive of survival benefits from intravenous BP therapy and are concordant with preclinical studies showing anti-myeloma activity. Current ASCO and European Myeloma Network guidelines recommend BP use in patients with MM and evidence of bone disease (10, 38) and are based exclusively on the bone-protective properties of BPs. The MRC Myeloma IX results now demonstrate BP benefits beyond bone protection—the reported anti-myeloma benefits of ZOL and possible bone-directed (SRE-reduction) benefits with initiating ZOL even before the development of overt bone disease provide a rational impetus to re-evaluate the role of BP therapy in patients with newly diagnosed MM. Indeed, these results are beginning to influence MM treatment guidelines. The UK Myeloma Forum updated its guidelines in September 2010 to advocate BP therapy for all patients with symptomatic MM regardless of bone lesion status and supports the preferential use of ZOL in MM (39). More recently, the NCCN amended its recommendations for BP use to include ‘all patients receiving primary myeloma therapy’ (11). Moreover, a Canadian expert panel issued a consensus statement in March 2011, supporting ZOL as the BP of choice for the treatment of myeloma bone disease and advocating further investigation into the use of ZOL as an anti-myeloma agent in light of the MRC Myeloma IX results (40).

It is notable that the earliest changes in BP recommendations for MM following publication of the MRC Myeloma IX results have come from regions where CLO is an established treatment option for patients with bone lesions from MM. In many regions, including the United States, PAM is extensively used to treat patients with MM, and haematologists in these regions might question whether the survival benefits observed with ZOL vs. CLO will be large enough to effect a change in treatment practices or not (i.e. vs. PAM) (41). Nonetheless, the MRC Myeloma IX results, together with ZOL effects seen in conjunction with bortezomib and other novel therapies (which may have independent effects on the course of myeloma bone disease), may yet further improve patient outcomes and provide an important platform to counter the effects of MM on bone health (15, 36, 37, 42).
